# Polyionic Tags as Enhancers of Protein Solubility in Recombinant Protein Expression

**DOI:** 10.3390/microorganisms6020047

**Published:** 2018-05-23

**Authors:** Vasiliki Paraskevopoulou, Franco H. Falcone

**Affiliations:** Division of Molecular Therapeutics and Formulation, School of Pharmacy, University of Nottingham, Nottingham NG7 2RD, UK; paxvp@nottingham.ac.uk

**Keywords:** protein solubility, peptide tag, protein fusion tag, polycationic, polyanionic, recombinant protein expression

## Abstract

Since the introduction of recombinant protein expression in the second half of the 1970s, the growth of the biopharmaceutical field has been rapid and protein therapeutics has come to the foreground. Biophysical and structural characterisation of recombinant proteins is the essential prerequisite for their successful development and commercialisation as therapeutics. Despite the challenges, including low protein solubility and inclusion body formation, prokaryotic host systems and particularly *Escherichia coli*, remain the system of choice for the initial attempt of production of previously unexpressed proteins. Several different approaches have been adopted, including optimisation of growth conditions, expression in the periplasmic space of the bacterial host or co-expression of molecular chaperones, to assist correct protein folding. A very commonly employed approach is also the use of protein fusion tags that enhance protein solubility. Here, a range of experimentally tested peptide tags, which present specific advantages compared to protein fusion tags and the concluding remarks of these experiments are reviewed. Finally, a concept to design solubility-enhancing peptide tags based on a protein’s pI is suggested.

## 1. Introduction

Since the first successful attempt at recombinant production of the human peptide hormone Somatostatin in *Escherichia coli* in 1976 [1], protein therapeutics have come a long way. Until then, small amounts of proteins and enzymes had to be extracted and purified from large amounts of animal or plant tissues, or from biological fluids. However, the revolution that came along with the recombinant production of proteins, enabling large-scale production of biological macromolecules, allowed commercial success in the biopharmaceutical industry [2].

The biochemical characterisation of proteins is of utmost significance and a prerequisite prior to their commercialisation. This process requires sufficient amounts of protein, which can be generated with the use of recombinant technology. Protein stability, three-dimensional structure in order to identify active sites, binding affinity for ligands or determination of their interaction are some of the features that are useful in protein characterisation. In addition, from a regulatory point of view, post-translational modifications, protein structure and propensity to aggregate have to be defined in the development process of biosimilars [3].

Many different host systems have been exploited for recombinant protein production, including various prokaryotic systems, yeast, insect, plant and mammalian cells [4]. Although prokaryotic systems lack the mechanisms for complex post-translational protein modification, such as glycosylation, and expression of complex protein folds involving disulphide bond formation can be challenging in these systems [5], they have certain characteristics that frequently make them the host system of choice for first-time protein production. In particular, *E. coli* is characterised by rapid growth at a low cost, with a cell doubling time of approximately 20 min [6]. This fast growth rate, in combination with the range of plasmids and safe, compatible strains that can be exploited, which can be flexibly tailored to the individual needs of recombinant production, makes this organism the ideal host [7].

## 2. Inclusion Bodies and Their Avoidance

Despite the clear advantages, recombinant expression of proteins in *E. coli* does not always guarantee success and is not obstacle-free, as there is not a single protocol that can be followed in order to avoid undesirable events. Insufficient yields, proteolytic degradation, protein misfolding, formation of inclusion bodies, as well as lack of protein or enzyme activity, are only a few of the possible unwanted outcomes [2].

**Figure 1:**
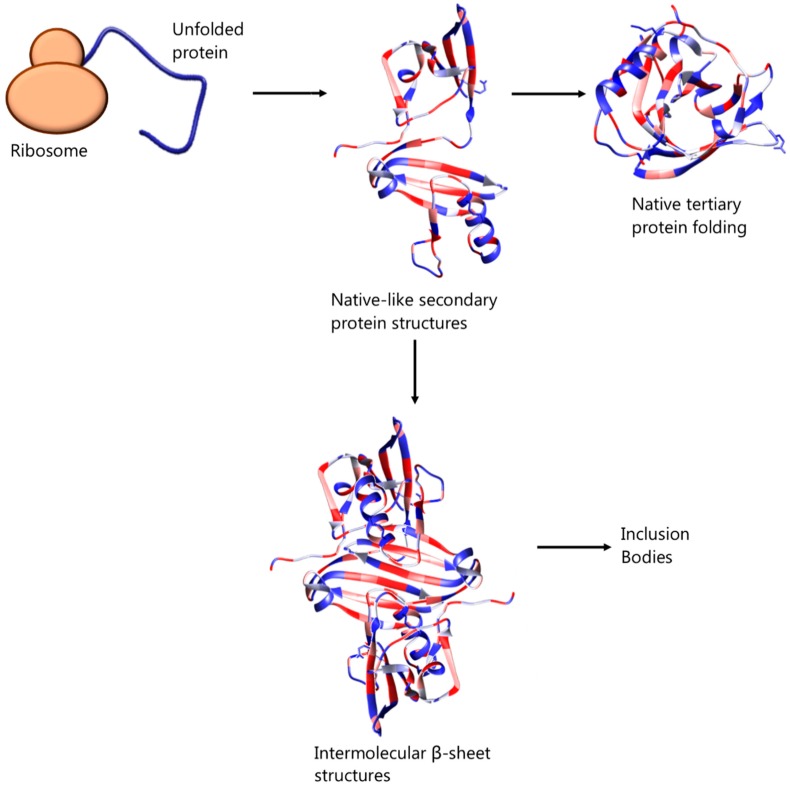
Suggested mechanism of native protein folding and association of intermediate, partially folded protein molecules, bearing native-like secondary structures, leading to inclusion body formation.

Previously thought to be the result of unspecific hydrophobic interactions among intermediate, partially folded, products of protein expression [8,9], inclusion bodies are now recognised as ordered, dynamic structures, the organisation of which depends on specific interactions. In particular, Fourier transform infrared microspectroscopy has revealed the presence of residual, native-like secondary structures and intermolecular β-sheet structures in bacterial inclusion bodies, which resemble the organisation of amyloid fibrils—see Figure 1 [10,11,12]. It has also been suggested that inclusion body formation can be caused by self-association of correctly folded protein of low solubility or unfolded protein molecules of mature protein [13]. In any case, the formation of inclusion bodies can represent a major drawback during heterologous or homologous proteins’ overexpression in bacterial host systems. This process is affected by and can be tuned with factors such as the environmental conditions (e.g., temperature, pH and ionic strength of culture medium), as well as the amino acid sequence of the protein [14] but can also be initiated by high protein concentration or inability of disulphide bonds to form in the reducing environment of the cytoplasmic space of the bacterial cells [13].

It should also be noted however, that more recently, formation of inclusion bodies has been seen as a potentially exploitable phenomenon, which has resulted in the development of strategies designed to enhance inclusion body formation, such as the addition of aggregation-prone tags or pull-down peptides [15,16]. Additionally, in some cases, the formation of inclusion bodies can facilitate protein purification. There are methods in place that allow the successful refolding of proteins from inclusion bodies, retaining their functionality [17]. However, the refolding process is not only time-consuming, but can also be problematic, yielding aggregated and/or inactive protein [18].

As Hippocrates taught, “prevention is better than cure.” Thus, various approaches aimed at preventing the formation of inclusion bodies, where undesirable, have been developed; these include optimisation of the growth conditions which lower the rate of protein expression and allow sufficient time for protein folding [19] and co-expression of molecular chaperones which mediate the correct folding of proteins [20,21].

Additionally, expression in the periplasmic space, where disulphide bonds can be formed in the presence of an oxidising environment [22], or mutations introduced in the reducing enzymes of the cytoplasmic space, which render them inactive and allow disulphide bond formation [23], have been exploited in order to overcome inclusion body formation. However, the most common approach is the fusion of the target protein with protein or peptide tags, which are known to enhance solubility [24]. One of the suggested mechanisms for the solubilisation effect is the increase of the net protein charge, which introduces repulsive electrostatic forces among protein molecules and promotes interaction with the solvent molecules [25,26].

A combination of the periplasmic expression approach with the addition of a peptide tag in the protein construct has been successfully used by our group in order to express a heterologous adhesin from *Helicobacter pylori* in *E. coli* [27].

## 3. Protein Fusion Tags

Although their mechanism of action has not been fully elucidated, protein fusion tags are very commonly used in order to enhance the solubility of recombinantly expressed proteins in *E. coli* [28]. One of the very first protein fusion tags used was a heterologous protein, glutathione S-transferase (GST), from the trematode *Schistosoma japonicum*, which has been employed for the production and purification of numerous proteins of mammalian origin in *E. coli* [29].

Another protein, thioredoxin (Trx), is thermally stable and can be overexpressed, retaining its solubility even at high concentrations. It has been employed in order to enhance the solubility and facilitate the expression of many mammalian cytokines and growth factors, previously contained in inclusion bodies. Attempts to explain how the fusion protein is resistant to forming inclusion bodies suggest that the highly soluble thioredoxin does not aggregate and allows time for correct folding of the fusion protein, among other reasons [30].

Maltose-binding protein (MBP), 42.5 kDa, a homologous *E. coli* protein used as a solubility- enhancing tag [31], is significantly larger than GST (26 kDa) and Trx (11.7 kDa)—see Table 1; however, out of the three, MBP demonstrated the biggest solubilisation effect, as well as chaperone-like behaviour [32].

Also, derived from yeast, a small ubiquitin-related modifier, or SUMO, has been found to have an even better solubilising effect than MBP [64]. Although its mechanism of enhancing solubility is currently unclear, it is speculated that it might act as a chaperone, similarly to Ubiquitin [65]. Alternatively, it might function as a nucleation point for the correct folding of the fusion protein [64].

In contrast to the above, it has also been reported that the introduction of MBP and Trx in the C-terminus of the mammalian proteinase procathepsin D did not prevent inclusion body formation but facilitated the recovery of soluble, yet not active, protein following refolding [66]. A few hypotheses regarding the solubility-enhancing mechanisms of protein fusion tags are described in [65]. These include the conformation of the fusion proteins into micelle-like structures, the attraction of chaperones or an intrinsic chaperone-like activity in the fusion proteins and the presence of electrostatic repulsive forces due to the protein’s net charge.

The solubilising effect of the protein tags seems to rely on the tags’ correct folding. However, due to their large size, their three-dimensional conformation can potentially interfere with the structure and most significantly with the activity of the expressed protein [67]. Thus, proteolytic removal of these tags after expression and purification of the fusion protein is common practice; however, the target protein’s solubility after tag removal cannot be predicted and the tag removal process might exert negative effects on the quality of the protein, such as product heterogeneity due to proteolytic cleavage at multiple sites, precipitation or poor recovery [24].

Although in most cases the removal of a big protein fusion tag is desired, there have been cases where the presence of the MBP has not proven an obstacle for the resolution of a crystal structure due to conformational heterogeneity attributed to the flexible linkage between the protein tag and the target protein [68]. In fact, techniques such as surface mutagenesis of MBP, in order to decrease entropy [69], or careful design of the linker between the MBP and the target protein [70], have been employed in order to facilitate crystallisation of the fusion protein.

## 4. Peptide Tags

As an alternative, small peptide tags have been used as solubility-enhancing tags, almost as early as protein fusion tags [71]. These peptide tags are relatively short, usually no longer than fifteen residues and comprise mostly one or two amino acids—repeated a varying number of times. They are polar and bear a positive or negative overall charge. Due to their small size and their repetitive amino acid content, they do not necessarily have an ordered three-dimensional conformation and are usually not resolved in protein crystal structures. This was the case for a hexalysine tag which was not defined in the crystal structure of the *Helicobacter pylori* adhesin BabA [72] or the ten different tags which were mostly invisible in [73]. As a result, they can exert their solubility-enhancing effect without interfering with the structure of the protein of interest or compromising its activity [67]. Additionally, an extra step for the removal of the peptide tags after production and/or purification is not necessarily required, in contrast to the case of protein fusion tags [24]. Finally, the expression of a large fusion protein tag instead of a short peptide tag is more demanding and acts as a metabolic burden on the bacterial hosts [25].

### 4.1. Polycationic Tags as Enhancers of Protein Solubility in Recombinant Protein Production

Since the first reference of a polylysine tag as a protein solubilising peptide tag in 1994 [71], many studies (reviewed here) have investigated the effect of different peptide tags on protein expression and solubility, without affecting the proteins’ function and activity. In this original study, a formerly chemically synthesised protein of low solubility, the minibody [74], was instead expressed in *E. coli* with a 3-lysine tag incorporated in either the N- or the C-terminus. As a result, the aqueous solubility of the tagged protein was increased by a 100-fold [71].

The introduction of two positively charged lysine residues in the N-terminus of the enzyme HemA [75] or of a hexalysine tag in the C-terminus of the protein BabA [27] led to the protection of the proteins against proteolytic degradation. Possible explanations for this stabilisation effect could be either the interference of the positively charged lysine residues preventing the binding of the proteases, or the correct folding of the tagged proteins [75]; a few proteases, such as DegP and Tsp, are known to show preference for mis- or unfolded target proteins, respectively. The hexalysine tag also seemed to strongly enhance the solubility of the recombinantly expressed BabA protein [27].

A slightly different solubilising tag, comprising glycine as well as lysine residues, proved to improve the solubility of the hydrophobic virus protein “u” (Vpu) from HIV-1, allowing its HPLC purification and 2D-NMR analysis in solution [76].

Other positively charged peptide tags were analysed for their effect on protein solubility, consisting of the basic amino acids arginine or histidine [25,67,77,78,79,80,81,82]. A comparison between arginine and lysine tags, from one to five residues long, in the N- or C-terminus of the poorly soluble bovine pancreatic trypsin inhibitor, revealed that the higher charges of the longer peptides had a bigger solubilising effect. Also, the position of the tag seemed to have an effect in this case, as the tags introduced to the C-terminus enhanced solubility more than the tags in the N-terminus of the protein. Finally, the arginine tags were more effective than the lysine tags of the same size in improving solubility, potentially due to the more hydrophilic character of arginine. The enhancement of the solubility was attributed to the repulsive electrostatic interactions between similarly charged tags, which prevent aggregation and allow sufficient time for correct folding, rather than their function as folding nuclei, which might have required a certain position in the expression construct [67].

Similar findings were obtained when positively charged arginine or lysine tags, comprising ten residues, were introduced into the enzyme *Candida antarctica* lipase B (CalB). The presence of these tags resulted in the transfer of the majority of the expressed protein from the insoluble to the soluble protein fraction in the cells, without affecting the protein expression yield overall [25].

As far as arginine tags are concerned, the introduction of a polyarginine tag in the C-terminus of the protein β-urogastrone, which led to the increase of the isoelectric point of the protein, has been used in order to facilitate the purification of the protein by cation exchange chromatography, which requires solubility in an aqueous system [77]. Recently, a C-terminal peptide tag rich in arginine was also exploited for the improved expression and enhanced solubility of the poorly soluble Tobacco Etch Virus (TEV) protease [78].

Histidine is the least basic amino acid out of the three, based on the pKa values of their side chains; 6.04 compared to 10.54 and 12.48 for lysine and arginine, respectively. Since the affinity of histidine-rich proteins for metal-ion resins was observed [83,84], hexahistidine tag has been established as one of the most popular affinity purification tags. The small size, the N- or C-terminal position that prevents interference with the function of the protein [85] and the highly selective interaction of histidine residues with nickel-NTA [86] are a few of the reasons that render the histidine tag so widely used in protein purification. However, when its effect on protein solubility was tested, it was found to be negative, resulting in lower protein solubility. In particular, the negative impact on protein solubility was stronger when the tag was found in the C-terminus, rather than the N-terminus, both in recombinant protein production in *E. coli* [79] and in a cell-free expression system [80].

Also, the effect of the length of a polyhistidine tag on protein expression was investigated and it was found that the longer decahistidine tag led to decreased expression yield of the protein aquaporin Z compared to the hexahistidine tag, without affecting the solubilisation of the protein by detergents [81]. Finally, the hexahistidine-tagged proteins were compared against proteins fused with other commonly used solubilising tags, such as GST and MBP and their relative solubility was found to be lower, as expected based on previous findings [82].

Chaperonins, a class of molecular chaperones that enhance protein folding in an ATP-dependent manner [87], have been found to interact with their substrate proteins based on the structural and biochemical properties of the latter. Hence, they can be classified based on their hydrophobic or polar interactions with protein substrates [88]. The molecular chaperonin CpkB from *Thermococcus kodakarensis* belongs to the second class, as it has been found that the negatively charged C-terminus of the enzyme facilitates its target protein recognition of positively charged proteins. The addition of a positively charged tag to the target protein (see Table 2) can lead to enhanced specificity of the negatively charged chaperonin for the target protein, mediated by attractive electrostatic interactions; this results in protection of the protein against thermal denaturation and appropriate folding [89].

It has also been reported in the literature that the activity of the chaperone Hsp90 to prevent aggregation and enhance correct protein folding entirely depends on two acidic regions, bearing negative charge; upon deletion of this charge, the anti-aggregation activity is compromised [90].

### 4.2. Solubilising Peptide Tags in Solid-Phase Peptide Synthesis (SPPS) and Native Chemical Ligation

The solubility enhancement effect of a polycationic tag has also been investigated in the fields of SPPS and native chemical ligation. A polycationic tag, rich in but not entirely consisting of arginine, introduced in both the N- and C-termini of poorly soluble peptides synthesised by Boc or Fmoc SPPS, rendered them soluble in water and allowed their purification in an aqueous environment [91,92,93]. The same effect and improved purification was observed after the addition of a pentalysine tag in the C-terminus of the poorly soluble A-chain of insulin glargine [94]. In all of the above examples, the solubilising tag was removed post purification.

In the case of native chemical ligation, which is a chemoselective reaction between an unprotected peptide with a C-terminal thioester modification and an also unprotected peptide with an N-terminal cysteine for the generation of native proteins [95], polyarginine tags, carrying positive charges, have been used. They have been shown to enhance the solubility of the peptide components of the membrane opioid receptor-like 1 [96] and the human immunodeficiency virus type 1 protease enzyme [97].

### 4.3. Polyanionic Tags as Enhancers of Protein Solubility in Recombinant Protein Production

Although so far positively charged polycationic tags which mostly enhance protein solubility have been reviewed, the opposite phenomenon has also been observed; polyanionic amino acid tags have been shown to enhance protein solubility too [98,99]. The addition of a negatively charged 9-aspartic acid tag led to increased solubility and expression of *Gaussia* luciferase in the soluble protein fraction [98]. Also, the presence of a polyaspartate tag resulted in increased protein expression and extracellular secretion of the periplasmic enzyme Asparaginase isozyme II [99].

Additions of single negatively charged residues, as well as longer sequences carrying negative net charge, were considered for their contribution to protein solubility of proteins prone to aggregation [100]. As with polycationic tags, the repulsive electrostatic interactions caused by the negative charge of the peptide tag seemed to enhance solubility and facilitate correct protein folding, by delaying protein aggregation, irrespectively of the size and structural conformation of the peptide tag [100]. Also, compared to commonly used solubility-enhancing fusion tags, such as MBP and Trx, peptide tags with high acidic content were found to enhance protein solubility to a greater extent [51].

### 4.4. Polycationic versus Polyanionic Tags

A few studies have compared homogeneous 5-amino acid long peptide tags comprising ten different amino acids with distinct biophysical properties (basic, acidic, polar and hydrophobic), side by side [73,101,102]. The overall conclusion of these studies was that the pentalysine tag had the biggest solubilisation effect, although the majority of the tags seemed to enhance solubility to a greater or lesser extent, excluding proline and isoleucine tags. It was revealed that although the positively charged peptide tags consisting of lysine or arginine led to increased solubility of bovine pancreatic trypsin inhibitor at two different pH values, 4.7 and 7.7, the acidic tags consisting of aspartic or glutamic acid, only improved solubility at pH 7.7, where their side chains were in their ionised state [73,101]. Also, from all of the aforementioned peptide tags, only the protein bearing a pentalysine tag was brought to high concentrations without reaching supersaturation and remained stable and aggregate-free at both pH values for up to two days [102].

A comprehensive list of polar, charged or neutral, peptide tags, which have been assessed and found to have a solubilisation effect on proteins that they are fused to, is presented in Table 2.

Following up on the observation that the majority of the signal peptides in the N-terminus of bacterial periplasmic proteins comprise basic amino acid residues, it was found that the presence of positive charges in the N-terminus is not essential for protein secretion [103]. To the contrary, when peptide tags with different biophysical properties were tested for their effect on protein expression and secretion of CalB, it was found that positively charged polylysine tags even hindered expression, while the negatively charged tags enhanced protein expression and secretion [104].

It has also been found that the presence of two arginine residues, bearing a positive charge, in the signal sequence of the mature protein alkaline phosphatase, restricted the secretion from the cytoplasmic to the periplasmic space; this could be either due to alteration of protein conformation or blockage of the secretion machinery when the positively charged N-terminus approaches the negatively charged phospholipids of the membrane [105]. Similarly, the presence of negative charge in the N-terminus leads to protein accumulation in the cytoplasm and delays protein secretion [103]. However, it has also been reported that this outcome can be reversed with the addition of a positively charged residue in the hydrophobic signal peptide [106].

It is worth mentioning that homogeneous polyionic tags, both positively and negatively charged, have been exploited in different applications in research. These include matrix-assisted refolding from inclusion bodies and protein purification, both mediated by the reversible immobilisation of tagged protein on an ion-exchange resin [107]. An example is the use of a polyanionic peptide tag consisting of varying number of glutamic acid residues for the purification of the polyoma coat proteinVP1 with anion-exchange chromatography [108].

This immobilisation feature can also facilitate the functionalisation of flat surfaces, by immobilising protein molecules on the surface in a specific and consistent orientation. Last but not least, the generation of chimeric bifunctional proteins, through the electrostatic attraction of two proteins with oppositely charged tags has been described; due to poor stability, however, the introduction of cysteine residues has also been studied for the formation of more stable, covalent disulphide bonds [107]. In particular, polyionic peptides have been exploited for the heterodimerisation of α-glucosidase fused with a 10-arginine tag and a modified Fab fragment fused with a 10-glutamic acid tag, both enhanced with a cysteine residue. The chimeric product retained both the enzymatic activity and antigen-binding capacity [109].

### 4.5. Polyionic Tags Displaying the Opposite Effect

It has also been reported in the literature that the presence of a hexalysine tag has led to recombinant protein production in inclusion bodies, due to the intramolecular attractive electrostatic interactions between the positively charged polylysine tag and the negatively charged protein at the intracellular pH 7.0 [26].

In addition, polypeptides comprising either lysine or glutamic acid residues have been exploited for the reversible precipitation of a range of proteins in low ionic strength solutions, which were then redissolved at physiological ionic strength (150 mM NaCl). Negatively charged proteins were precipitated by mixing with polylysine peptides and positively charged proteins were precipitated by mixing with polyglutamic acid peptides. The cause of precipitation is the intermolecular attractive electrostatic interactions between the proteins and the free peptides [110].

## 5. Supercharging of Proteins

Based on all of the aforementioned, it is also important to consider the effect of the overall protein charge on solubility, not found localised in one of the two termini but spread across the protein sequence and surface. So, although it is known that proteins are least soluble at their isoelectric points where they do not bear any net positive or negative charge, it was desired to prove that protein charges prevent aggregation [111]. The mutation of positively charged arginine residues of eukaryotic proteins expressed in *E. coli* to negatively charged aspartate residues resulted in enhanced protein solubility [37]. Increased solubility was also observed following mutation of residues of green fluorescent protein exposed to the solvent to positively or negatively charged residues, leading to highly charged protein molecules [111].

This process, called supercharging, prevented both thermally and chemically induced protein aggregation [111]. The same effect of enhanced solubility and stability was also observed after supercharging a human enteropeptidase; it was speculated that a small increase in the protein’s net charge by supercharging the protein resulted in more significant increase in protein solubility than the solubility enhancement conveyed by peptide tags [112]. Nonetheless, the point mutations involved in the supercharging of protein surfaces could result in loss of protein activity and/or alter its biochemical properties [73].

## 6. Discussion

As explained above, there are several factors that can render a protein insoluble or lead to the formation of inclusion bodies during recombinant protein production; not only high protein concentrations can result in aggregation but also large proteins are more prone to it. Of course, the composition of a protein in its primary amino acid sequence is crucial for its propensity to aggregate, as long hydrophobic regions will make the protein less soluble [113].

On the other hand, protein net average charge, as well as hydrophilicity, are known to be related to protein solubility [114]. The average charge of a protein at a certain pH value depends on the pI, which can be calculated from the pKa values of the side chains of the residues that are ionised. Due to protein folding, the experimental pKa values, hence the pI of the protein, can be slightly different from the calculated value. As mentioned earlier, proteins are the least soluble at their pIs and their solubility increases at differing pH values [115].

**Figure 2:**
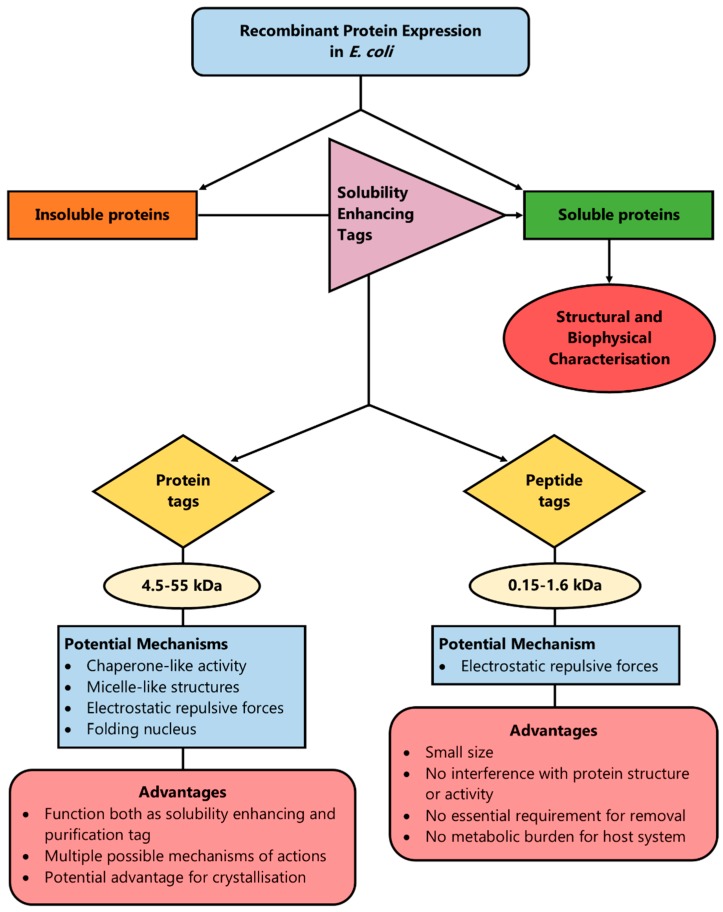
A method to enhance protein solubility during recombinant protein production is the introduction of solubility-enhancing tags (protein or peptide) in the recombinant plasmid. By having a few potential mechanisms of action, protein tags can cover a wider range of proteins in order to enhance solubility and most of them act simultaneously as solubility and purification tags. However, peptide tags are more versatile and smaller in size, which means their removal is not always essential, they do not pose a burden on the host system’s metabolism and they do not affect the target protein’s structure or function.

Thus, the introduction of net charge by the addition of even a single amino acid residue can enhance solubility by introducing repulsive electrostatic interactions between protein molecules that allow sufficient time for the correct folding of proteins, or by disrupting hydrophobic interactions between or within the same protein molecule [25]. Such peptide tags amplify the solubility properties of the amino acids regardless of their position in the N- or C-terminus of the protein, which offers the advantage of flexibility [66]. Another advantage of peptide tags over protein fusion tags is that in order to exert their solubilisation effect, an ordered secondary structure is not required, as is the case for the protein fusion tags, in the cases when they potentially function as a folding nucleus [101]. The above rationale has been summarised in Figure 2.

A method, based on highly conserved amino acid sequences in a range of soluble proteins, has been described for the design of novel solubility-enhancing peptide tags [116]. It is suggested that the solubility of a protein can be theoretically calculated and controlled. However, it is acknowledged that there always needs to be compatibility between the protein of interest and the solubility controlling peptide tag [116].

## 7. Conclusions

From all the above, it becomes obvious that the choice of the most appropriate solubility-enhancing tag depends on the individual protein and requires careful design; generalisation should be avoided [64]. Peptide tags have overall benefits compared to the protein fusion tags, due to their small size and versatility [73]. It is speculated that the introduction of a peptide tag bearing similar charge as the protein of interest at a certain pH value in either of the protein’s termini will enhance solubility due to inter- and intramolecular repulsive interactions. Peptide tags of the opposite charge to the protein of interest should be avoided, as they could lead to protein precipitation instead.

## Figures and Tables

**Table 1:** Protein fusion tags for solubility enhancement during recombinant protein production.

Name	Full Name	Size (kDa)	Reference
GST	Glutathione-*S*-transferase	26	Smith et al., 1988 [29]
MBP	Maltose-binding protein	42.5	Maina et al., 1988 [31]
UB	Ubiquitin	~9	Butt et al., 1989 [33]
Trx	Thioredoxin	11.7	LaVallie et al., 1993 [30]
Z-tag/ZZ-tag	IgG-binding domain from protein A	15.5/31	Samuelsson et al., 1994 [34]
GB1	Immunoglobulin-binding domain of protein G	6.2	Huth et al., 1997 [35]
DsbA	Disulphide isomerase I	21.1	Collins-Racie et al., 1998 [36]
DsbA^mut^			Zhang et al., 1998 [37]
NusA	N-utilization substance A	55	Davis et al., 1999 [38]
IF2 domain I (or *InfB*(1-471)	Initiation factor 2		Sorensen et al., 2003 [39]
CaBP	Calcium binding protein		Reddi et al., 2002 [40]
SUMO	Small ubiquitin-related modifier	31	Malakhov et al., 2004 [41]
FTN-H	Ferritin heavy-chain		Ahn et al., 2005 [42]
Skp	Seventeen kilodalton protein	17	Chatterjee et al., 2006 [43]
T7PK	T7 protein kinase	4.5	Chatterjee et al., 2006 [43]
Ecotin	*E. coli* trypsin inhibitor	16	Malik et al., 2006 [44]
RpoA	RNA Polymerase α-subunit	39.5	Ahn et al., 2007 [45]
PotD	Spermidine/putrescine-binding periplasmic protein	39.8	Han et al., 2007 [46]
Crr	Glucose-specific phosphotransferase (PTS) enzyme IIA component	20	Han et al., 2007 [46]
Tsf	Elongation factor Ts	30.6	Han et al., 2007 [47]
SlyD	Aggregation-resistant protein	22.2	Han et al., 2007 [48]
msyB	Acidic protein	14	Su et al., 2007 [49]
RpoS	RNA polymerase sigma factor	39	Park et al., 2008 [50]
yjgD		15	Zou et al., 2008 [51]
rpoD	σ 70 factor of RNA polymerase	20	Zou et al., 2008 [51]
HaloTag7	Inactive derivative of DhaA, a bacterial haloalkane dehalogenase	34	Ohana et al., 2009 [52]
*sf*GFP	Superfolder green fluorescent protein		Wu et al., 2009 [53]
Mocr	Monomeric bacteriophage T7 0.3	16.7	DelProposto et al., 2009 [54]
SNUT	Solubility eNhancing Ubiquitous Tag	19	Caswell et al., 2010 [55]
EspA	*E. coli* secreted protein A	25	Cheng et al., 2010 [56]
ArsC	Arsenate reductase	16	Song et al., 2011 [57]
BLA	AmpC-type β-lactamase		Tokunaga et al., 2010 [58]
*InfB*1-21	Entity of *InfB*(1-471) responsible for increased expression	28	Hansted et al., 2011 [59]
Fh8	*Fasciola hepatica* antigen	8	Costa et al., 2014 [28]
SmbP	Small metal-binding protein	9.9	Vargas-Cortez et al., 2016 [60]
Ffu	β-fructofuranosidase truncations	17.7–29.5	Cheng et al., 2017 [61]
TDX	Tetracopeptide domain-containing thioredoxin	35	Xiao et al., 2018 [62]
HE-MBP(Pyr)	Truncated maltotriose-binding protein with modified histidine tag		Han et al., 2018 [63]

**Table 2:** Polyionic or polar peptide tags assessed for their solubility enhancement effect during recombinant protein expression.

Name	pI	Size (kDa)	Reference
**Polycationic tags**			
(Arg)_1–5_	10.00–12.62	0.174–0.799	Kato et al., 2007 [67]
(Arg)_5_ (His)_5_	12.62 7.66	0.799 0.704	Islam et al., 2015 [73] Islam et al., 2012 [101] Khan et al., 2015 [102]
(Arg)_10_	12.95	1.6	Jung H-J et al., 2011 [25] Johnson et al., 2007 [97]
(Arg-Gly-Gly)_3_-Gly	12.30	0.886	Englebretsen et al., 1999 [92]
Poly(Arg)			Smith et al., 1984 [77]
(Gly-Arg)_3_-(Arg)_3_	12.91	1.5	Kalpana et al., 2018 [78]
(Gly-Arg)_4_	12.48	0.871	Englebretsen et al., 1996 [91]
Gly-(Arg)_5_	12.62	0.856	Sato et al., 2005 [96]
Gly(Arg-Gly-Gly)_3_ Gly(Lys-Gly)_6_	12.30 10.70	0.886 1.2	Choma et al., 1998 [93]
(Gly)_2_-(Arg)_2_-Gly-Arg Gly-Lys-Gly-(Lys)_2_	12.30 10.28	0.658 0.517	Gao et al., 2017
(Gly)_2_(Lys)_4_	10.47	0.645	Park et al., 2003 [76]
(Lys)_1–5_	8.88–10.61	0.146–0.659	Kato et al., 2007 [67]
(Lys)_2_	10.00	0.274	Wang et al., 1999 [75]
(Lys)_3_	10.28	0.402	Bianchi et al., 1994 [71]
(Lys)_5_	10.61	0.659	Islam et al., 2015 [73] Hossain et al., 2009 [94] Islam et al., 2012 [101] Khan et al., 2015 [102]
(Lys)_6_	10.70	0.787	Hage et al., 2015 [27]
(Lys)_10_	10.94	1.3	Englebretsen et al., 1999 [92]
**Polyanionic tags**			
(Asp)_5_	3.34	0.593	Kim et al., 2015 [99] Kim et al., 2014 [104]
(Asp)_5_ (Glu)_5_	3.34	0.664	Islam et al., 2015 [73] Islam et al., 2012 [101] Khan et al., 2015 [102]
[Gly-(Asp)_3_]_3_	3.16	1.2	Rathnayaka et al., 2011 [98]
Negative peptide extensions (>−6)			Zhang et al., 2004 [100]
**Polar tags**			
(Asn)_5_ (Gln)_5_ (Ser)_5_	5.50 5.50 5.50	0.588 0.659 0.453	Islam et al., 2015 [73] Islam et al., 2012 [101] Khan et al., 2015 [102]
